# Use of New Technologies in the Prevention of Suicide in Europe: An Exploratory Study

**DOI:** 10.2196/mental.7716

**Published:** 2017-06-27

**Authors:** Juan-Luis Muñoz-Sánchez, Carmen Delgado, Andrés Sánchez-Prada, Mercedes Pérez-López, Manuel A Franco-Martín

**Affiliations:** ^1^ Complejo Asistencial de Zamora Department of Psychiatry Zamora Spain; ^2^ Universidad Pontificia de Salamanca Salamanca Spain

**Keywords:** suicide, suicide attempt, self-harm, prevention, new technologies, Europe

## Abstract

**Background:**

New technologies are an integral component of today’s society and can complement existing suicide prevention programs. Here, we analyzed the use of new technologies in the prevention of suicide in 8 different European countries.

**Objective:**

The aim of this paper was to assess the opinions of professionals in incorporating such resources into the design of a suicide prevention program for the region of Zamora in Spain. This investigation, encompassed within the European project entitled European Regions Enforcing Actions against Suicide (EUREGENAS), includes 11 regions from 8 different countries and attempts to advance the field of suicide prevention in Europe.

**Methods:**

Using a specifically designed questionnaire, we assessed the opinions of 3 different groups of stakeholders regarding the use, frequency of use, facilitators, content, and format of new technologies for the prevention of suicide. The stakeholders were comprised of policy and public management professionals, professionals working in the area of mental health, and professionals related to the social area and non-governmental organizations (NGOs). A total of 416 participants were recruited in 11 regions from 8 different European countries.

**Results:**

The utility of the new technologies was valued positively in all 8 countries, despite these resources being seldom used in those countries. In all the countries, the factors that contributed most to facilitating the use of new technologies were accessibility and free of charge. Regarding the format of new technologies, the most widely preferred formats for use as a tool for the prevention of suicide were websites and email. The availability of information about signs of alarm and risk factors was the most relevant content for the prevention of suicide through the use of new technologies. The presence of a reference mental health professional (MHP) was also considered to be a key aspect. The countries differed in the evaluations given to the different formats suggesting that the cultural characteristics of the country should be taken into account.

**Conclusions:**

New technologies are much appreciated resources; however they are not often underused in the field of suicide prevention. The results of this exploratory study show that new technologies are indeed useful resources and should be incorporated into suicide prevention programs.

## Introduction

Suicide is a severe public health problem and one of the most common forms of unnatural deaths worldwide [1]. Globally, around 800,000 people commit suicide every year and it is estimated that for every person who commits suicide another 20 people have attempted to do so [2]. During the second half of the last century, suicide was one of the 3 main causes of death in people in the 15 to 44 age group [3]. Despite this, suicide rates are not stable over time and they show short-term variations and trends [4]. Currently, the mean rate of suicide worldwide is 11.6 cases per 100,000 people [5], and there are substantial country-specific differences around the world, with greater differences observed between culturally different populations [6].

Overall, Europe has a high rate of suicide, but the epidemiology of suicide varies among the different countries [7]. Some countries, such as Finland, Hungary, and the Baltic countries, together with Russia and Belarus, have the highest suicide rates in the world, with 40 suicides per 100,000 people. By contrast, countries in the south of Europe such as Italy, Spain, and Greece, have low levels [8]. Although Spain is among the European countries with the lowest rates of suicide, suicide levels have increased considerably in recent years. According to data from the National Institute of Statistics (NIS), suicide is the first cause of unnatural deaths in Spain, and in 2012, the number of suicides increased by 11.3%, the highest rate recorded since 2005 [9].

The economic and human costs of suicidal behavior are very high for the individuals involved, including their families and society in general. In the United States, it has been estimated that deaths due to suicide cost the country around US $26 billion per year in medical costs and absence from work [10].

Suicidal behavior is a complex phenomenon consisting of biological, clinical, psychological, and social factors [11]. Research has shown that some characteristics that are crucial for evaluating the risk of suicide can be identified [12] and that these risk factors can provide early signals as well as pathways for preventive interventions aimed at reducing the probability that a person will attempt to commit suicide [13]. Suicide is tightly linked to the model of the society in which an individual lives, there being a direct relationship between the experience of stress factors or unfavorable alterations in a person’s environment and the risk of suicide [14-16]. It has been reported that inhabiting an environment with good living conditions and without economic hardships decreases the risk of suicide [17-19]. For example, divorced individuals have a greater risk of suicide [20]. On the other hand, religion is generally a protective factor such that the degree of religiousness is indirectly proportional to suicide risk [21,22].

Suicidal acts are usually preceded by “softer” manifestations such as thoughts of death or suicidal ideation [23]. The progression from thought to actually committing suicide represents the transition from a slight symptomatology to a more severe one [24]: prodromic symptomatology is a risk factor for future admission to hospital or a factor of risk of death by suicide [25]. Many studies, both clinical and community-based, have reported consistent empirical evidence that the presence or history of mental illness is the greatest risk factors for suicide in the general population [26], with mood swings, the loss of control over impulsive behavior, alcohol and substance abuse, psychotic ,and personality disorders being responsible for the highest risks of suicide and suicidal behavior [27]. It has been estimated that between 80% and 95% of people who commit suicide, including adolescents and elderly persons, have some kind of psychiatric disturbance [28]. Of all psychiatric diseases, affective disorders, and in particular recurrent major depressive disorder (MDD), are those that involve the greatest risk of suicide in both men and women in almost all age ranges [29]. Epidemiological studies have suggested that 15% of individuals with recurrent MDD have attempted to commit suicide at some time in their lives [30].

Suicide and suicide prevention are attracting increasing attention worldwide [31]. The act of committing suicide impacts all levels of society and results in an increase in the risk of attempts at suicidal behavior by others in the environment surrounding the person who dies. Suicide should thus not be considered an individual problem, but rather a problem that affects that person’s family, his or her surroundings, and society in general. Accordingly, it is crucial to seek a strategy aimed at preventing suicide at the public health level and not focused exclusively on the individual level [32]. Further, suicide is tightly linked to other forms of violence and health problems [33]. Over the past 20 years, public health systems have attempted to calculate suicide rates, identify the risk factors and protective factors, and have tried to develop effective strategies for preventing suicide. However, a significant amount of work remains to be done in these areas; one of the emerging challenges for public health systems is how to determine the ways of disseminating and putting into practice “what we know” about the prevention of suicide on a large scale in order to achieve an impact at a demographic level [10]. As such, it has been proposed that to carry out programs aimed at preventing suicide it is imperative to be knowledgeable about the people involved with and related to suicide.

Stakeholders are individuals, groups, or organizations that participate directly in decision making and actions [34] and many groups have demonstrated the importance of stakeholders in the design of strategies for intervention in the field of general clinical practice [35], mental health [36], and more specifically, in the field of suicide prevention [37].

New technologies are an integral component of today’s society and are under constant development and expansion. There are many contexts in which new technologies play a relevant role and their use in the health field is expanding [38], especially in the area of mental health [39,40]. The aim of this paper was to (1) assess the opinions of stakeholders from different European countries regarding the use of new technologies for the prevention of suicide, such as informative websites, online self-help interventions, electronic therapy (e-therapy) interventions, interactive websites (chats), Internet forums, social networks, and apps; and (2) assess their opinions in incorporating such resources into the design of a suicide prevention program in Zamora, Spain. This investigation, encompassed within the European project entitled European Regions Enforcing Actions against Suicide (EUREGENAS), included 11 regions and attempted to promote the field of suicide prevention in Europe [41].

## Methods

### Participants and Procedure

Within the context of the EUREGENAS project, our study aimed at evaluating—on a European scale—the actions to be implemented and considered effective in the prevention of suicide. The objective was to determine the different points of view and the possibilities of introducing these actions. Beginning with a first consultation with the partners involved in the project and an in-depth review of the literature, a list of possible stakeholders of interest was proposed. The 3 main categories of stakeholders established were: (1) decision policy makers (DPM), stakeholders in the political and public management field; (2) mental health professionals (MHP), stakeholders working in the area of mental health; and (3) professionals related to the social area and those working in non-governmental organizations (NGOs) (NGOs/Social Area) (Multimedia Appendix 1).

A total of 416 participants were recruited in 11 regions from 8 different European countries according to the following inclusion criteria: (1) workers belonging to the 3 professional groups selected for this study (DPM, MHP, NGOs/Social Area); (2) experienced in the field of suicide; (3) aged between 18 to 65 years old.

### Variables and Instruments

Specific questionnaires were tailored to the various stakeholders and were used as tools to obtain the information necessary for assessing needs. The questionnaires included closed questions about the use of new technologies for the prevention of suicide and sociodemographic data (gender, age, professional category). The questionnaires were created by project partners, and subsequently revised by all the members of the project. They were drafted originally in English and translated into the mother tongue of the various project partners. The projects partners distributed approximately 60 questionnaires each and were administered face-to-face or via email.

A total of 416 questionnaires were completed (Table 1). The gender distribution was 39.7% (165/416) men and 60.3% women (251/416). With respect to age, 61.8% (257/416) were aged between 40 and 59 years, 26.8% (111/416) between 20 and 39 years, and 11.4% (48/416) were over 60 years of age.

**Table 1:** Questionnaires administered by country (N=416).

Country	Stakeholders, n (%)			
	DPM^a^	MHP^b^	NGO^c^	Total
Belgium	14 (3.4)	19 (4.6)	15 (3.6)	48 (11.5)
Finland	7 (1.7)	21 (5.0)	31 (7.5)	59 (14.2)
Germany	9 (2.2)	9 (2.2)	12 (2.9)	30 (7.2)
Italy	10 (2.4)	13 (3.1)	9 (2.2)	32 (7.7)
Romania	10 (2.4)	19 (4.6)	3 (0.7)	32 (7.7)
Slovenia	10 (2.4)	11 (2.6)	9 (2.2)	30 (7.2)
Spain	17 (4.1)	92 (22.1)	45 (10.8)	154 (37.0)
Sweden	10 (2.4)	13 (3.1)	8 (1.9)	31 (7.5)
Total	87 (20.9)	197 (47.4)	132 (31.7)	416 (100.0)

^a^DPM: decision and policy maker.

^b^MHP: mental health professional.

^c^NGO: non-governmental organization.

### Statistical Analyses

The data obtained from the questionnaires were analyzed using the SPSS v19 software package. Once the data was gathered, and depending on the study objectives, comparisons of means were performed to gain a general idea of the scores obtained on the different items. After this first descriptive analysis, multivariate analysis of variance (MANOVA) was implemented to determine the existence of significant differences. Finally, using multidimensional scaling (ASCAL), we sought to visually recognize the dimensional patterns in the preferences of the training formats both by country and by the participants involved in the investigation.

## Results

### Utility and Frequency

**Figure 1:**
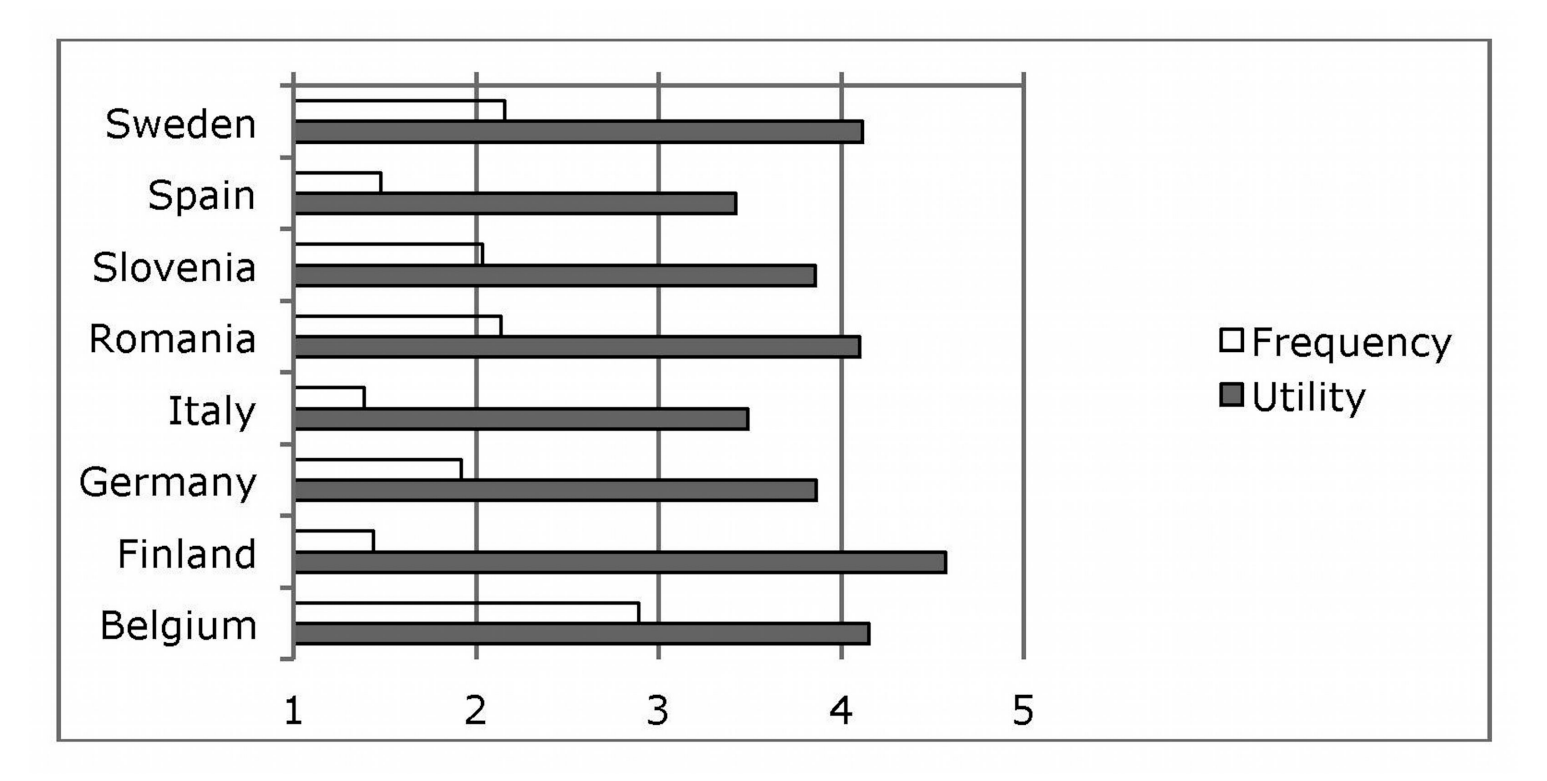
Country-specific differences in utility and frequency.

The utility of the new technologies evaluated from 1 (not very useful) to 5 (very useful) and was judged positively in all the countries with a mean (SD) of 3.93 (0.78). However, the frequency of use, evaluated from 1 (never) to 5 (always), was low with a mean (SD) of 1.79 (1.08). Belgium was the only country that approached a moderate frequency (Figure 1).

**Figure 2:**
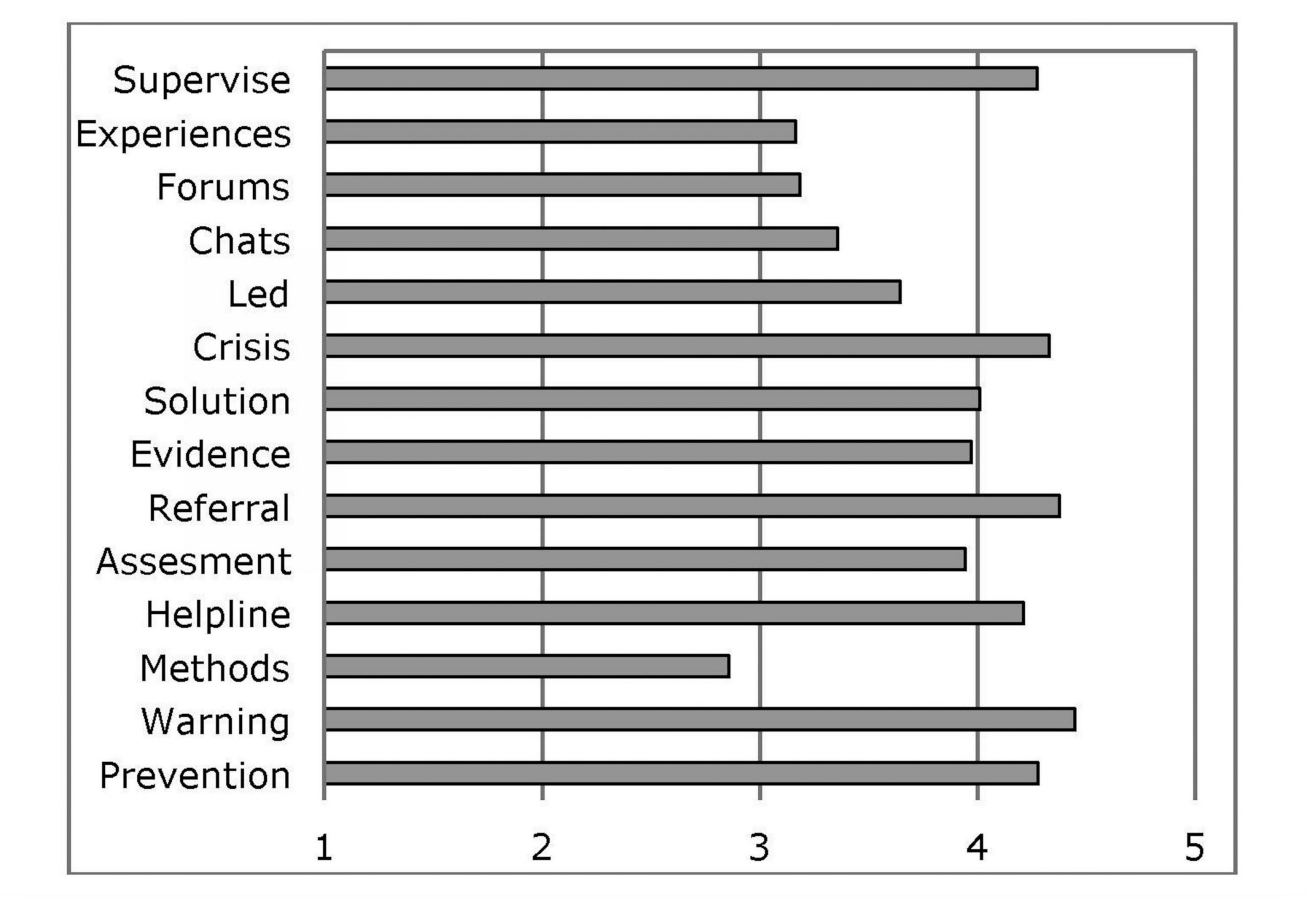
Relevant content for the prevention of suicide with new technologies.

MANOVA failed to reveal significant differences among stakeholders with regards to utility (*P*=.138) or frequency of use (*P*=.141). In contrast, there were significant differences between the countries, both regarding utility (*P*<.001) and frequency of use (*P*<.001). Finland, Sweden, Belgium, and Romania considered the technologies to be more useful than Spain (*P*<.01). Belgium used them the most frequently while Finland, Spain, and Italy used them the least.

### Facilitators

The elements that would facilitate the use of new technologies for the prevention of suicide (Textbox 1) were assessed on a scale ranging from 1 (not at all) to 5 (absolutely).

**Elements that would facilitate the use of new technologies.**

Element

Training: more information about the issue through training

Newsletters: more information about the issue through informative bulletins

Automated: automated apps (ie, those that do not require constant supervision)

Accessible: ease of access

Anonymity: guaranteed anonymity

Time: saving time

Cost: saving money

Free: no additional costs (freeware)

It was determined that accessible was the most important element (4.15), followed by free (4.12), anonymity (3.91), training (3.77), time (3.67), cost (3.51), newsletters (3.23), and finally automated (3.16). These factors were judged differently by the various stakeholders (Pillai’s trace test *P*<.001). MHPs found training more importance than DPMs (*P*<.001). In contrast, DPMs attributed more importance to cost than the MHPs (*P*<.001). NGOs attributed intermediate values between both DPMs and MHPs, with no significant differences. The elements also had country-specific differences (Pillai’s trace test *P*<.001). For example, Romania valued training the most, Sweden valued accessible the most, whereas Slovenia valued newsletters, automated, and time the most.

### Relevant Content for the Prevention of Suicide

The following content were evaluated on a scale ranging from 1 (not necessary) to 5 (absolutely necessary): (1) prevention (information about the prevention of suicide); (2) warning (information about the warning signs and risk factors); (3) methods (information about how people commit suicide); (4) helpline (online help links for the prevention of suicide) (5) assessment (scales of risk assessment); (6) referral (referral to a professional and/or organization); (7) evidence (evidence-based therapy); (8) solution (offer solutions to the people at risk of committing suicide); (9) crisis (crisis contingency plans in cases of high suicide risk); (10) led (chats guided by a professional); (11) chats (chats and Internet support forums); (12) forums (Internet chats and forums for therapeutic uses); (13) experiences (exchange of experiences between people at risk of committing suicide); and (14) supervise (supervision by a professional).

**Figure 3:**
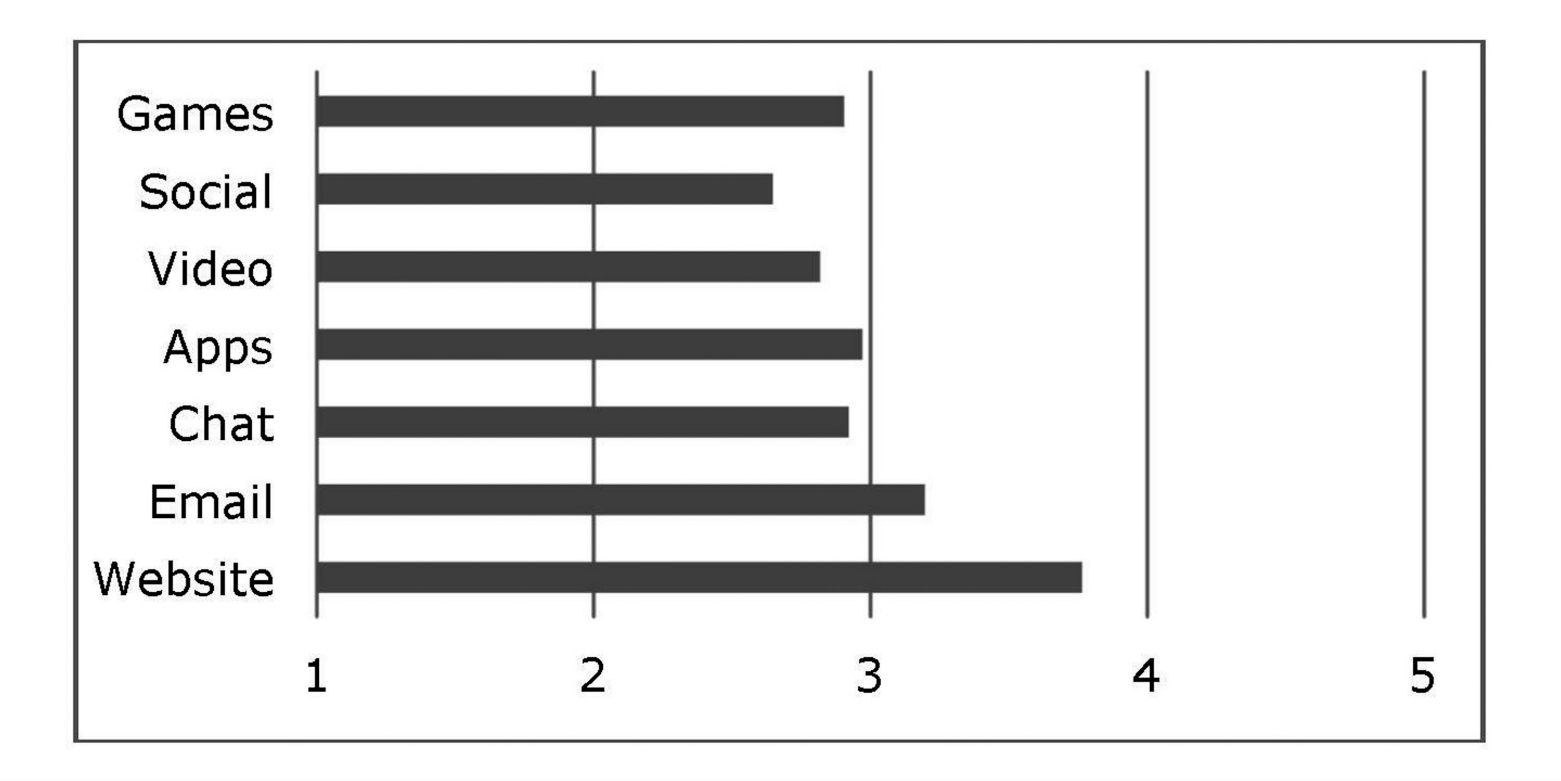
Format-specific preferences of new technologies.

With the exception of methods, the evaluation was positive (>3). The content with the best evaluations were warning, referral, crisis, supervision, and prevention. The least well-evaluated were experiences and forums (Figure 3).

Statistically significant differences were found among the stakeholders (Pillai’s trace test *P*<.001). DPMs attributed less value to helpline than MHPs (*P*=.004) and the NGOs (*P*=.001). They also gave more importance to referral than NGOs (*P*=.012). In contrast, MHPs attributed less importance than DPMs to led (*P*=.019) and to chats (*P*=.008).

**Figure 4:**
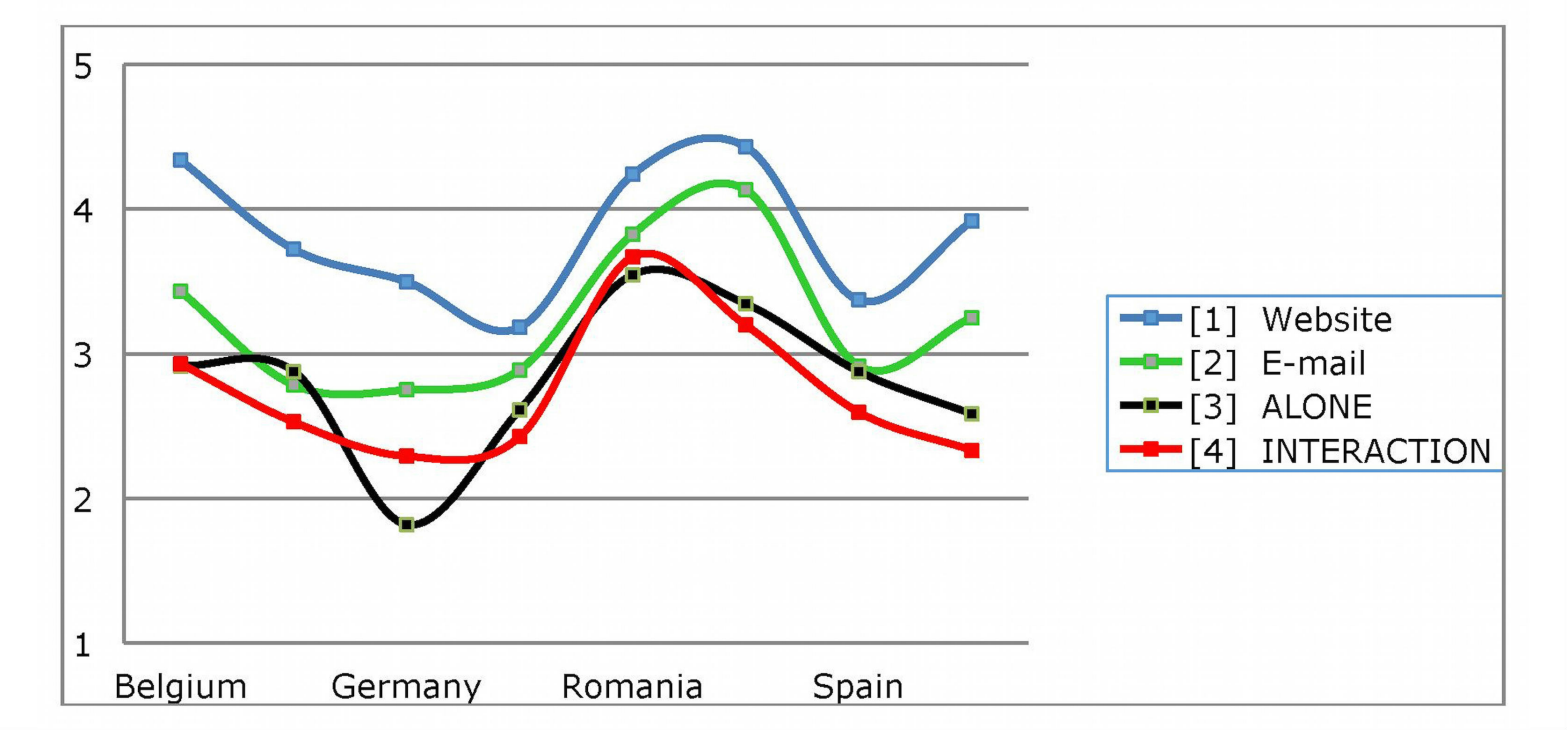
Country-specific format preferences.

Statistically significant differences were also observed between countries (Pillai’s trace test *P*<.001). Pairwise comparisons revealed differences in the importance of some content (*P*<.01). Specifically, Italy had the lowest evaluations for all of the content except evidence, which was evaluated less by Finland. Solution was evaluated less well by Belgium, whereas supervise was evaluated less well by Sweden. The highest evaluations corresponded to Romania and Slovenia in all the content except assessment and referral, which were better evaluated by Finland and Belgium, respectively.

### Preferred Formats

Formats were also assessed on a scale ranging from 1 (never) to 5 (always). The mean evaluations are shown in Figure 3. Website was the most preferred format, followed by email. The other formats did not reach a frequent intention of use. In addition, a MANOVA comparison did not reveal significant differences among the stakeholders for any of the formats (Pillai’s trace test *P*=.468), although there were differences among countries (Pillai’s trace test *P*<.001). Slovenia gave the highest evaluations to games, social networks, email, and website, while Romania scored video, apps, and chat the highest.

ASCAL was implemented to explore structure in the preferences for the various formats. Two underlying dimensions were detected that permitted the identification of 2 differentiated criteria in format preference. The fitting of the data to these dimensions was excellent (stress=.04; r square = .988). The following differentiated types were detected: (1) website (focused on personalized information); (2) email (focused on personal and/or individual communication); (3) games, videos, and apps (focused on activities that do not require interaction and can be done alone [ALONE]); and (4) social networks and chats (focused on activities that do require social interaction [INTERACTION]).

Website was determined to be the most preferred, with a mean (SD) value of 3.76 (1.22), followed by email, with a mean (SD) of 3.20 (1.26). Finally, no differences were found between ALONE and INTERACTION, with mean (SD) values of 2.94 (1.12) and 2.80 (1.20), respectively.

No differences were detected regarding the preference for format types (Pillai’s trace test, *P*=.134). In contrast, there were country-specific differences (Pillai’s trace test, *P*<.001) (Figure 4).

Pairwise comparisons (Bonferroni correction, *P*<.05) revealed that website was more preferred by Slovenia, Belgium, and Romania, than Italy and Spain. Email was more preferred by Slovenia and Romania than by Finland. ALONE was more preferred by Romania and Slovenia than by Germany, and INTERACTION was more preferred by Romania than by all the other countries.

## Discussion

### Principal Findings

It is known that new technologies are increasingly important in daily life, especially among young people. This has led companies, policy makers, and other stakeholders to use them more frequently in order to access their target population and achieve their aims. Notwithstanding, within the sphere of public health the use of such technologies is still in its infancy, especially in the case of suicide and its prevention. There is evidence that suggests the probable benefit of Web-based strategies in suicide prevention [42]. In this sense, the findings of the present study confirm their scant application as all of the countries ranked them as rarely, with the exception of Belgium that ranked them as sometimes. Despite this, the results of this exploratory study suggested that the use of new technologies for the prevention of suicide could be well-accepted among the various stakeholders. As such, utility was assessed positively in all the countries included in the study, with Finland evaluating it the highest, whereas Spain and Italy, although still positive, assessed it the lowest. These findings confirmed the cultural differences with regards to both the use of new technologies and the problem of suicide, since northern European countries had a more positive view of the use of new technologies than countries in the south of Europe [43]. This may be correlated to the degree of implementation of new technologies in each of the respective countries and their use in public health services.

All the evidence suggests a need to promote suicide prevention programs based on new technologies that will serve to gain better access to the younger sector of society. It is clear that new technologies can be a tool that complements existing suicide prevention programs; the view of stakeholders, from the areas of education, health, and social and legal affairs, is that they are an instrument to be developed and tested. In a recent review, Robert and colleagues affirmed that the Internet is useful for linking people who feel lonely or isolated, can provides access to suicide prevention information and resources, and can influence vulnerable people to attempt suicide, but it can also be used to prevent self-harm and suicide [44].

Taking into account the possibility of developing technological applications to improve the prevention of suicidal behavior, we analyzed the factors that would facilitate a more generalized use of new technologies. In general, all the proposals made were evaluated positively as factors, such that promoting the more widespread use of these technologies could offer a broad solution in which to act, emphasizing the accessibility and availability of free software. In light of the results, such promotion should be focused and dependent on the receivers. For example, in the case of mental health workers, training should be stressed in order to palliate the lack of knowledge and/or availability of resources to implement the new technological applications in this field. By contrast, in the case of MPS, it would be of greater interest to stress the low costs of the resources. These data are consistent with our findings in that countries in which they are considered to have greater applicability, it is most important to foster accessibility and use, whereas in countries in which their applicability is not considered so highly, it is more important to focus on training. As a result, it is necessary to promote training, especially in European countries, and increase accessibility at a country level. To achieve this, the European Union should make efforts to offer a global space of communications to facilitate these developments. Recently, de Beurs and colleagues showed the efficacy of an electronic learning (e-learning)- supported “Train-the-Trainer” program. This program would be an effective strategy for implementing clinical guidelines and improving care for suicidal patients [45].

Another important aspect to be taken into account is the applications should be developed with the use of new technologies. For example, those involving the following were considered to be of greatest interest: warning, prevention, supervise, crisis, referral, and helpline, as compared with other content proposed. These observations showed that the most important contribution of the use of new technologies was linked to the monitoring of persons at risk of suicide and providing them with the opportunity to access attention. In this sense, helpline, warning, supervise, and crisis scored the highest. It should be noted that epidemiological data are currently allowing the identification of populations at risk of engaging in suicidal behavior; that specific treatments are available, and that perhaps the best contribution of new technologies lies in their providing the opportunity to monitor and intervene rapidly in this at-risk population when a critical situation occurs. It is also necessary to consider that there are differences in the appreciations of technologies between the various stakeholders. MHPs confer greater importance to referral than DPMs, which may be explained by their being able to access or attend to this at-risk population. On the other hand, DPMs gave more importance to led and chat than MHPs because they may value the positive effect of mutual support. The mental health network, which has the capacity and the obligation to carry out group interventions, psychoeducation, and pharmacological treatment when there is an associated psychiatric disorder, values as more relevant the ability to detect cases of very high risk or cases in crisis, and that under such circumstances, the person can be referred to a mental health center. A meta-analysis of computer-based psychological treatments for depression shows the efficacy and effectiveness of such treatments in diverse settings and with different populations [46]. By contrast, in other prevention resources, or for professionals working in prevention, more importance is given to the social function of the new technologies. In this sense, they are not counterpoised elements, even though from the care-taking point of view it appears that the detection and monitoring of limit cases are elements to be incorporated into the applications so that they will be well-accepted by health professionals, especially professionals working in the field of mental health. This, however, does rule out applications that favor social relations and even direct contact with the user. Likewise, differences are also seen among countries, although they may be due to the high evaluation levels in most of the content given by Slovenia and Romania in comparison with the other countries.

Finally the formats website and email were the ones most highly valued. The other formats received a low evaluation, with social networks the least well valued. The differences among countries again place Slovenia and Romania as the countries that ranked website and email the highest, as opposed to Italy and Germany, which ranked them the lowest. These findings may be related to the most widely used formats, and hence, are considered of greater utility than the other formats, which, although with increasing penetration into society, especially among young people, are not considered as relevant, at least in the initial stages. Several studies have evaluated the effectiveness of Web-based interventions for suicidal thoughts [47-49]. As such, it is important to consider the type of formats that emerge in ASCAL analyses. The underlying structure allowed us to identify 4 format types: (1) website (oriented more towards information); (2) email (oriented more towards personal and/or individual communication); (3) ALONE (oriented more towards resources that can be used alone, such as games, videos, and apps); and (4) INTERACTION (oriented more towards social interaction via chats or social networks).

There are thus 2 criteria that should be taken into account. The first compares the resources based on the solitary/interactive nature of their use. With respect to solitary use, website and ALONE are resources that users can make use of when alone and they do not require interaction with other people. On the other hand, interactive use (email and INTERACTION), were characterized by the fact that users must interact with other people. In the case of email, the interaction is personalized while in the case of social networks it collective. In either case, the user must be able to perform such interactions. The second criterion compares resources that require greater activity by users with resources that demand less activity. In the first case, one would be dealing with ALONE-type activities, which demand a sufficient level of activity to be able to watch a video on YouTube, become involved in a video game, or download an app with the purpose of gaining greater efficiency in the use of the technology. Social network or chat (INTERACTION) resources would also be included within this set of resources that require a certain level of activity to become engaged in group interactions. Alternatively, resources that do not require the same level of activity, either because they are individualized interactions (not collective, since they are real or virtual), such as communication by email or the search for information via websites. A striking observation in the results was that greater importance was given to the simplicity of the resource, especially in northern European countries, as compared with the socialization or not of the resources. In this sense, the impact of simplicity is less in countries in the south of the continent such as Italy, Spain, and even Romania, and to some extent Slovenia, than in northern countries since in the former the scores were very close for all the options.

These resources suggest a specificity that should be taken into account in their adaptations for the different aims for which they can be used. It is likely that some of them may be better used than others for certain purposes or types of user. When comparing the evaluations by the stakeholders, no differences between them were seen; however, they did receive country-specific evaluations. These differences possibly reflect diversity in the more or less communicative character of the cultures as well as in the value of social interaction in the various countries studied. Accordingly, the peculiarities of each country should be taken into account in order to design programs that incorporate the resources that best match the social psychology of the users to which they are directed. It is interesting that Germany is the country that most values the use of resources that facilitate socialization and interaction. It is also striking that in all the countries, websites were considered to be the most widely accepted resource. This could be attributed to the search for simplicity or as the first step taken when a situation arises.

### Limitations and Strengths

The questionnaires used to collect the data were generated internally by the members of the project and did not take into account psychometric criteria. The principal aim of the project was to analyze the knowledge of relevant professionals in the suicide field to improve and create prevention programs of suicide in different regions of Europe. It should be noted that the questionnaire were not designed to be a tool used in the prevention of the suicide, rather were made as data compilation tools. In addition, the questionnaires were not translated in a homogenous way but were translated by different project partners, using different resources for the various languages.

The stakeholders involved in the study were not randomly selected and thus do not represent stakeholders as a whole. The number of stakeholders involved in the study differed per country, as well as did their motivation for participating. The sociodemographic data collected in the questionnaires (gender, age, and professional category) could have impacted the findings, but was not possible to control because of the small study sample size. However, the goal of the study was to make a first assessment of the usefulness of new technologies in prevention approaches for suicide. From this point of the view, the results of the study must be interpreted from a qualitative standpoint. In all cases, the stakeholders were selected following the same criteria and were persons involved direct or indirectly with suicide and the consequences of it. Therefore, in all cases, their opinions were derived from their knowledge about this problem. Indeed, involving diverse stakeholders to try to reach a consensus is increasingly well-accepted as the future of collaborative, influential research [50].

Taking into account these limitations, the differences between countries can be associated to different perspective of the specific stakeholders selected instead of proper general differences between countries. However, the data may be used to better understand the possibilities and potential benefits of the use of new technologies in suicide prevention. To our knowledge, this if the first study examining country-specific differences in Europe about this topic.

### Conclusion and Clinical Implications

The results of this exploratory study showed that new technologies are useful resources that can offer possibilities in the field of suicide prevention. We found new technologies to be well-accepted and well-valued by the various stakeholders (MHPs, DPMs, and NGOs). As such, they should be used in suicide prevention programs. Placing greater importance on resources that are accessible, free, can guarantee anonymity, incorporate training for mental health professionals, and reduce the time required for suitable management through automation, would facilitate and possibly increase the use of these resources.

## Appendix

Multimedia Appendix 1 Categories and subcategories of stakeholders.

## Abbreviations

ASCAL: multidimensional scaling
DPM: decision and policy maker
EUREGENAS: European Regions Enforcing Actions against Suicide
MANOVA: multivariate analysis of variance
MDD: major depressive disorder
MHP: mental health professional
NGO: non-governmental organization
