# Supplementary material for: Use of New Technologies in the Prevention of Suicide in Europe: An Exploratory Study
Source: JMIR Ment Health. 2017 Jun 27;4(2):e23. doi: 10.2196/mental.7716 (PMC5506332; doi:10.2196/mental.7716)
Supplement: Multimedia Appendix 1 [file mental_v4i2e23_app1.pdf]

**Category**	**Subcategory**
**1. Decision and Policy Makers**	1a. European networks focusing on mental health promotion.
	1b. Decision and policy makers from local and regional authorities (dealing with mental health, care, welfare, family matters, youth…).
	1c. Decision and policy makers in public health institutions (eg, mental health care centers, hospitals).
	1d. Private companies influencing policy (eg, health insurance).
	1e. Media.
	1f. Educational setting, policy makers.
	1g. Professionals working in financial services and human resources.
**2. Mental Health Professionals (youth, adult and elderly focused MHPs)**	2a. General practitioners.
	2b. Psychologists (inpatient, outpatient).
	2c. Psychiatrists (inpatient, outpatient).
	2d. Emergency physicians (on call doctors in Accident & Emergency).
	2e. Nursing staff who work with suicidal patient (primary health nurse, mental health nurse, emergency room nurse).
	2f. Rescue personnel (paramedic – ambulance crew).
	2g. Work setting, e.g. private companies and prevention advisors in occupational medicine.
	2h. Educational setting, eg, schools, school counselors.

**3. NGOs/ Social Area**	3a. Professionals in the social area (community social workers, home help workers, youth workers, social welfare services).
	3b. Staff of NGOs and agencies working in the following areas: youth, marital counseling, family and life counseling, welfare.
	3c. Educational setting: teachers.
	3d. Staff of suicide helplines.
	3e. Representatives of religious group.
	3f. Support groups with survivors.
	3g. Work setting: employers, human resources, union representatives.
	3h. Criminal justice stakeholders (eg, police, penitentiary police, coroners…).
	3i. Pharmacists.
